# Cholecystocolonic Fistula: A Case of Chronic Diarrhoea and Hidden Stones

**DOI:** 10.7759/cureus.73129

**Published:** 2024-11-06

**Authors:** Brecht Hens, Hendrik Reynaert

**Affiliations:** 1 Gastroenterology and Hepatology, Universitair Ziekenhuis Brussel, Brussels, BEL

**Keywords:** acute cholangitis, chronic diarrhoea, colonoscopy, gallstone cholecystitis, laparoscopic cholecystectomy complication

## Abstract

A cholecystocolonic fistula (CCF) is a rare cause of chronic diarrhoea. It most often occurs in elderly women as a result of chronic inflammation due to gallstone disease or, rarely, malignancy. Curative treatment consists of cholecystectomy with excision of the fistula tract, but it is often overlooked preoperatively and thus entails a higher risk of postoperative complications. Here, we present a case of a 78-year-old woman with chronic diarrhoea who was diagnosed with a CCF during a colonoscopy. Cholecystectomy was complicated by acute cholangitis due to an obstructive stone in the common bile duct (CBD) that was masked preoperatively due to alternative biliary drainage via the CCF. Recognition of this rare entity can enhance clinicians’ diagnostic appraisal and limit postoperative complications.

## Introduction

Chronic diarrhoea, commonly defined as the presence of an increased stool frequency, urgency, or loose stools for at least four weeks, frequently poses a diagnostic challenge to clinicians [1]. Its differential diagnosis is broad, and common causes include drug-induced diarrhoea, irritable bowel syndrome, coeliac disease, and inflammatory bowel disease. Contrary to acute diarrhoea, chronic diarrhoea in immunocompetent individuals is usually not caused by an infectious agent. A thorough history and physical examination are therefore essential and can guide further use of laboratory or imaging studies [2].

A cholecystocolonic fistula (CCF) is a rare cause of chronic diarrhoea and is often diagnosed during rather than before cholecystectomy. Most commonly described in women, its clinical spectrum is diverse, with chronic diarrhoea likely resulting from bile acid malabsorption, as its predominant symptom.

Here, we describe a case of chronic diarrhoea caused by a CCF, diagnosed during colonoscopy and complicated by acute cholangitis after cholecystectomy.

## Case presentation

A 78-year-old woman was referred to the endoscopy clinic by her primary care physician because of diarrhoea and a 7- to 8-kg weight loss in the last three months. She denied a history of rectal blood loss, abdominal pain, loss of appetite, fever, or recent visits abroad. Her past medical history was notable for melanoma in situ on her upper back, which was resected two years prior to the current presentation. The physical examination was unremarkable. General blood tests, including a complete blood count, kidney and liver function tests, and analysis of faecal samples, including bacterial cultures and microscopy for parasites, were normal.

**Figure 1:**
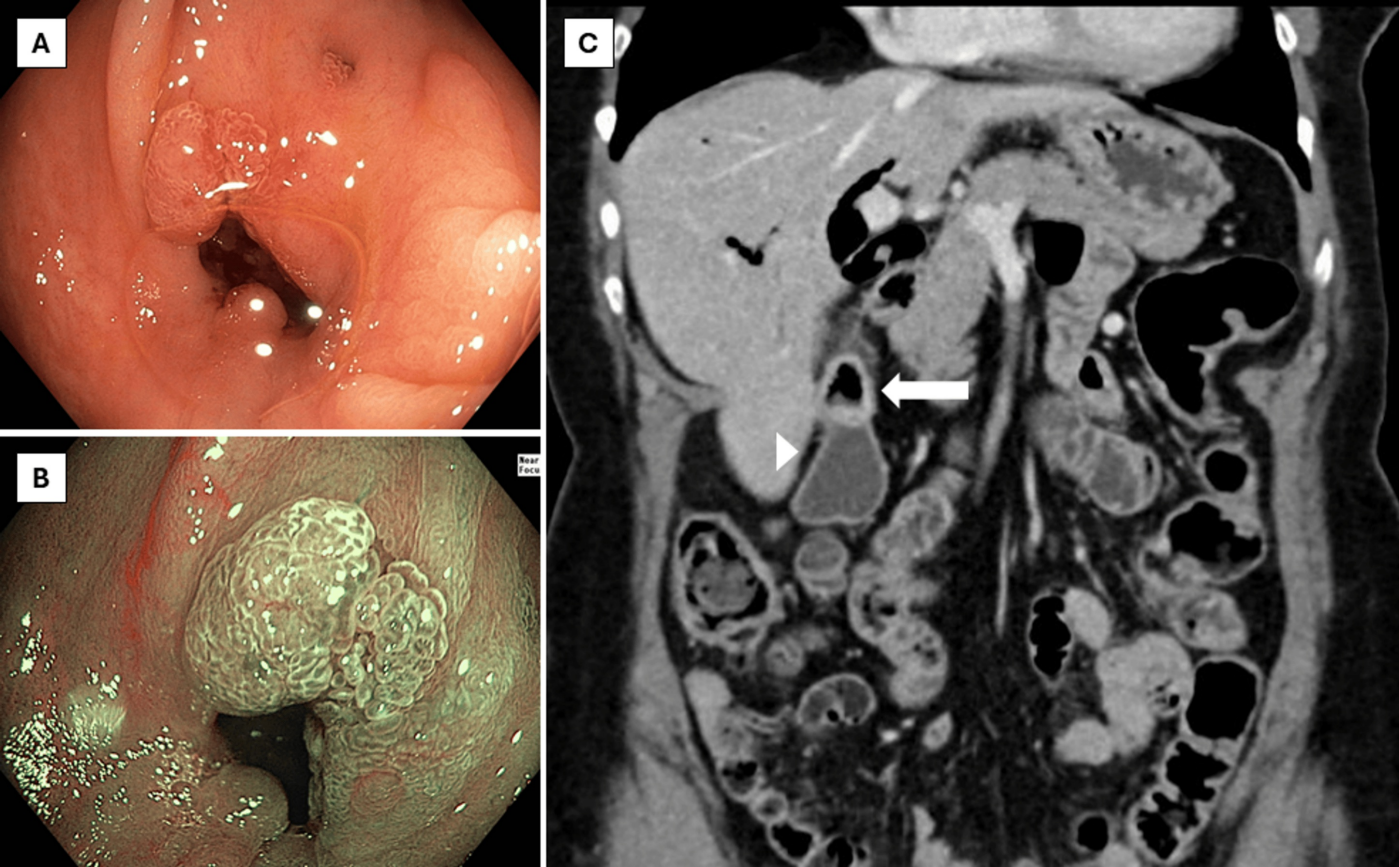
Initial colonoscopy and CT Panel A: White light endoscopy showing a diverticulum-like defect in the ascending colon. Panel B: Virtual chromoendoscopy (narrow band imaging, Olympus) of the defect. Panel C: Computed tomography (CT) with intravenous contrast (coronal view) showing close contact between gallbladder (arrow) and ascending colon (arrowhead) with pneumobilia.

Ileocolonoscopy showed a normal mucosa of the terminal ileum and colon, apart from 1 diminutive adenomatous polyp in the sigmoid colon and diverticula throughout the colon. In the ascending colon, however, there was a diverticulum-like defect with tissue proliferation extending from the inside towards the borders of the defect with a chicken skin appearance of the surrounding mucosa (Figure 1). Endoscopic imaging with high-definition white light and virtual chromoendoscopy suggested reactive changes rather than a neoplastic process based on the vascular pattern. Biopsies from the aberrant area showed regenerative tissue, and random biopsies from the right and left colon excluded microscopic colitis. A contrast-enhanced computed tomography (CT) of the abdomen, which was performed a couple of days after the colonoscopy, showed marked pneumobilia with fistulisation between the ascending colon and gallbladder with signs of chronic cholecystitis. No radiopaque gallstones were described (Figure 1).

**Figure 2:**
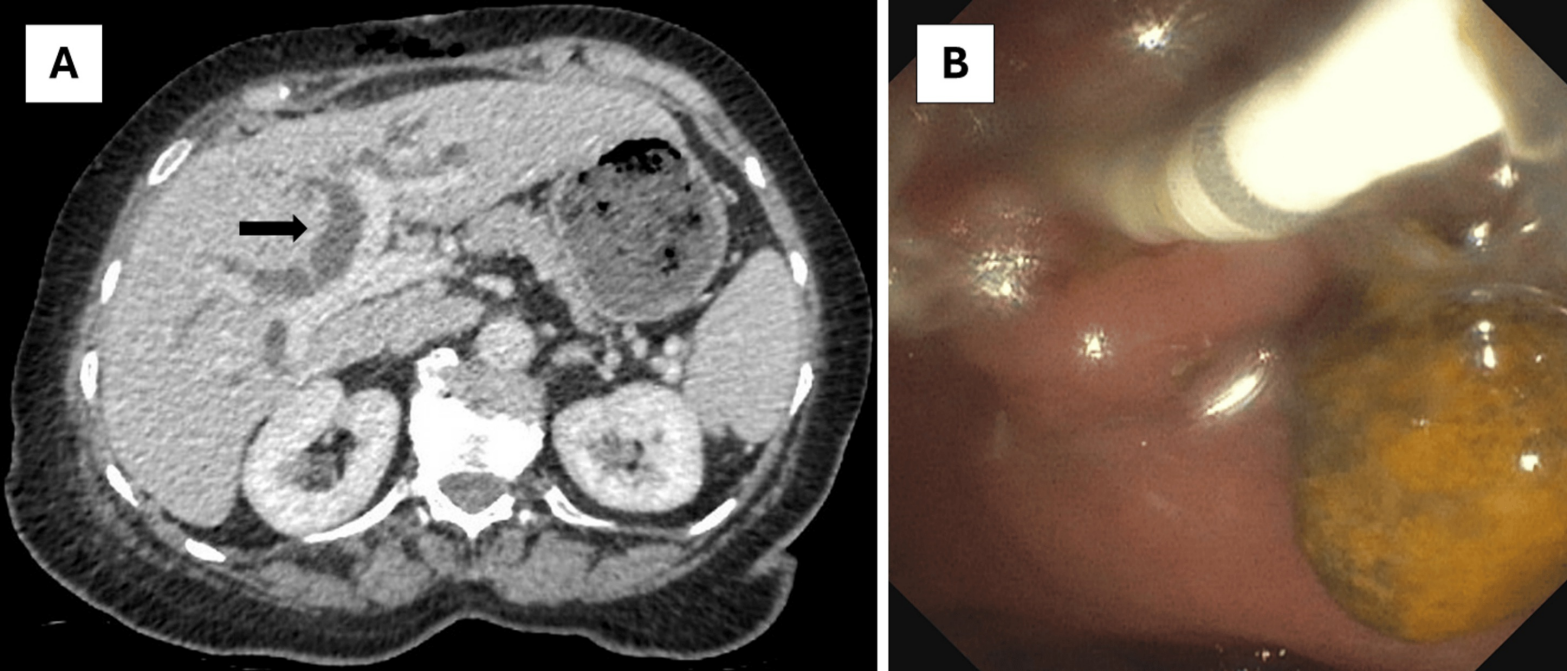
Postoperative CT and ERCP Panel A: Computed tomography (CT) with intravenous contrast showing marked dilatation of the intrahepatic bile ducts (arrow). Panel B: View during endoscopic retrograde cholangiopancreatography (ERCP) after sphincterotomy and removal of the obstructing gallstone.

A diagnosis of chronic diarrhoea due to a CCF was made, and the patient was referred for cholecystectomy. One month later, she underwent a laparoscopic cholecystectomy with wedge resection of the fistula complex. The resection specimen showed chronic cholecystitis without dysplasia or carcinoma and confirmed the presence of a CCF. The patient was discharged in a stable condition the next day but was readmitted two days later because of fever, pronounced cholestasis, and dilatation of intra- and extrahepatic bile ducts on a CT performed at the emergency department (Figure 2). Intravenous ceftriaxone was started for acute cholangitis, and the patient was booked on the next available endoscopic retrograde cholangiopancreatography (ERCP) list two days later. This revealed a large stone in the common bile duct (CBD), which was extracted after sphincterotomy with spontaneous drainage of purulent liquid afterwards (Figure 2). The patient had a swift clinical recovery and was discharged home two days later. Follow-up at the outpatient clinic one month after surgery showed marked improvement in diarrhoea and quality of life.

## Discussion

In the last decades, CCFs have been increasingly recognised as a cause of chronic diarrhoea [3]. Less frequent than cholecystoduodenal fistulas, these fistulas are estimated to occur in one out of 1000 patients undergoing cholecystectomy. They are rarely diagnosed preoperatively, potentially exposing patients to an increased risk of postoperative complications and higher conversion rates from laparoscopic approaches to laparotomy [4,5].

Multiple diagnostic modalities have been described in the literature, but studies directly comparing diagnostic accuracy are lacking. In our case, the combination of colonoscopy and contrast-enhanced CT resulted in the diagnosis of a CCF. In a recent retrospective single-centre study in patients with a cholecystoenteric fistula, the use of transabdominal ultrasound and CT resulted in a preoperative diagnosis in 31.0% of patients and in 2/4 of patients with CCF. In 80% of patients, an indistinct border between the gallbladder and adjacent gastrointestinal structures was identified on imaging, which should raise the suspicion of a cholecystoenteric fistula [6]. While reports on ERCP’s diagnostic accuracy vary, it can be valuable, achieving a 63% accuracy rate in some studies [7].

The clinical spectrum is diverse, and symptoms, if any, are typically present for several months before diagnosis. Women are more frequently affected than men, with a median age at diagnosis of 68.9 years [8]. The predominant symptom is chronic diarrhoea, likely resulting from bile acid malabsorption due to disruption of the enterohepatic cycle by leakage of bile acids directly into the colon [8]. The clinical triad of pneumobilia, chronic watery diarrhoea, and vitamin K malabsorption is considered pathognomonic for CCF [9]. In contrast to cholecystoduodenal fistulas, the high bacterial load in the colon also increases the risk of infectious complications in CCFs, manifesting as right upper quadrant pain or recurrent bouts of cholangitis. Acute complications such as gallstone ileus or massive bleeding are rare [8].

Formation of CCFs is considered a consequence of chronic cholecystitis; however, other factors, such as gallbladder cancer, have also been associated [8,10]. In this case, it is thought that the intra-gallbladder pressure increased due to cholecystitis, causing a fistula to form between the ascending colon and the gallbladder. It is likely that the gallstone became impacted in the CBD only after the formation of the fistula so that bile flow was diverted to the colon, masking the presence of this stone and preventing cholangitis preoperatively.

The management of patients with CCF should be patient-tailored depending on the presence or absence of symptoms or complications and comorbid conditions. Clear guidelines are lacking, but cholecystectomy with excision of the fistula tract is generally considered the treatment of choice. Traditionally, open cholecystectomy combined with excision and closure of the fistula was advised [3,8]. Taking into account a higher rate of conversion to laparotomy and a variably longer operating time, minimally invasive approaches have been increasingly used in experienced centres and have shown to be feasible, safe, and associated with rapid postoperative recovery [6,11]. However, nonoperative strategies can be effective in selected patients and should be considered in those unfit for surgery. Several reports on patients with CCF and biliary obstruction show that biliary decompression by endoscopic sphincterotomy can lead to symptom resolution and may induce healing of the fistula [12-14].

Cholecystectomy, in this case, was complicated by acute cholangitis, likely due to a CBD stone already present preoperatively. In 2019, the American Society for Gastrointestinal Endoscopy published a management algorithm for patients with symptomatic cholelithiasis. This algorithm recommended a risk-stratification approach to determine the need for preoperative biliary evaluation based on clinical predictors [15]. Cholangiography was deemed unnecessary in patients at low risk, but additional imaging such as endoscopic ultrasound (EUS) or magnetic resonance cholangiopancreatography (MRCP) was advised for intermediate-risk patients (i.e. abnormal liver tests or age >55 years or dilated CBD on ultrasound), as was preoperative ERCP for high-risk patients (i.e. bilirubin >4 mg/dL and dilated CBD, or cholangitis, or a visible CBD stone on imaging). The alternative biliary drainage in case of a CCF can easily mask the presence of CBD stones and decrease the accuracy of these clinical predictors. In addition, the sensitivity of CT for the detection of cholelithiasis is limited as not all stones are radiopaque, and many are isodense with bile [16,17]. Even though our patient did not recall episodes of biliary colic or cholecystitis, had normal liver function tests, and did not have a dilated CBD or a radiopaque stone on imaging, she would have benefited from EUS or MRCP prior to cholecystectomy. 

## Conclusions

A CCF is an uncommon cause of chronic diarrhoea. Cholecystectomy with excision of the fistula tract is the treatment of choice, but the postoperative complication rate might be higher, and caution should be taken to exclude stones or masses in the biliary tract preoperatively, as their presence may be masked by the alternative drainage of the gallbladder. Therefore, preoperative EUS or MRCP should be considered to exclude biliary obstruction and prevent postoperative complications.
